# Cell-of-origin classification using the Hans and Lymph2Cx algorithms in primary cutaneous large B-cell lymphomas

**DOI:** 10.1007/s00428-021-03265-5

**Published:** 2022-01-14

**Authors:** Anne M. R. Schrader, Ruben A. L. de Groen, Rein Willemze, Patty M. Jansen, Koen D. Quint, Tom van Wezel, Ronald van Eijk, Dina Ruano, Cornelis P. Tensen, Esther Hauben, F. J. S. H. Woei-A-Jin, Anne M. Busschots, Anke van den Berg, Arjan Diepstra, Maarten H. Vermeer, Joost S. P. Vermaat

**Affiliations:** 1 Department of Pathology, Leiden University Medical Center, P.O. Box 9600, 2300 RC Leiden, The Netherlands; 2 Department of Hematology, Leiden University Medical Center, Leiden, The Netherlands; 3 Department of Dermatology, Leiden University Medical Center, Leiden, The Netherlands; 4 Department of Pathology, University Hospitals Leuven, Leuven, Belgium; 5 Department of General Medical Oncology, University Hospitals Leuven, Leuven, Belgium; 6 Department of Dermatology, University Hospitals Leuven, Leuven, Belgium; 7 Department of Pathology, University Medical Center Groningen, Groningen, The Netherlands

**Keywords:** Primary cutaneous diffuse large B-cell lymphoma, Leg type, Primary cutaneous follicle center lymphoma, Cell-of-origin, Hans algorithm, Lymph2Cx algorithm

## Abstract

Primary cutaneous diffuse large B-cell lymphoma, leg type (PCDLBCL-LT) and primary cutaneous follicle center lymphoma with a diffuse population of large cells (PCFCL-LC) are both primary cutaneous B-cell lymphomas with large-cell morphology (CLBCL) but with different clinical characteristics and behavior. In systemic diffuse large B-cell lymphoma, not otherwise specified (DLBCL-NOS), gene-expression profiling (GEP) revealed two molecular subgroups based on their cell-of-origin (COO) with prognostic significance: the germinal center B-cell-like (GCB) subtype and the activated B-cell-like (ABC) subtype. This study investigated whether COO classification is a useful tool for classification of CLBCL. For this retrospective study, 51 patients with PCDLBCL-LT and 15 patients with PCFCL-LC were analyzed for their COO according to the immunohistochemistry-based Hans algorithm and the NanoString GEP-based Lymph2Cx algorithm. In PCFCL-LC, all cases (100%) classified as GCB by both Hans and Lymph2Cx. In contrast, COO classification in PCDLBCL-LT was heterogeneous. Using Hans, 75% of the PCDLBCL-LT patients classified as non-GCB and 25% as GCB, while Lymph2Cx classified only 18% as ABC, 43% as unclassified/intermediate, and 39% as GCB. These COO subgroups did not differ in the expression of BCL2 and IgM, mutations in *MYD88* and/or *CD79B*, loss of *CDKN2A*, or survival. In conclusion, PCFCL-LC uniformly classified as GCB, while PCDLBCL-LT classified along the COO spectrum of DLBCL-NOS using the Hans and Lymph2Cx algorithms. In contrast to DLBCL-NOS, the clinical relevance of COO classification in CLBCL using these algorithms has limitations and cannot be used as an alternative for the current multiparameter approach in differentiation of PCDLBCL-LT and PCFCL-LC.

**Supplementary Information:**

The online version contains supplementary material available at 10.1007/s00428-021-03265-5.

## Introduction

Primary cutaneous diffuse large B-cell lymphoma, leg type (PCDLBCL-LT) and primary cutaneous follicle center lymphoma with a diffuse population of large cells (PCFCL-LC) are both primary cutaneous B-cell lymphomas with large-cell morphology (CLBCL), but with different clinical characteristics and behavior [1]. PCDLBCL-LT is an aggressive lymphoma with a 5-year disease-specific survival (DSS) of 56% despite standard immunochemotherapy (R-CHOP). PCFCL-LC usually has an indolent behavior with a 5-year DSS of 95% and is preferably treated with local radiotherapy [1, 2]. PCDLBCL-LT was initially recognized as a subgroup of PCFCL-LC with a somewhat different morphology, typical presentation with skin lesions on the leg(s), and a more unfavorable prognosis [3]. In 1997, this resulted in recognition of PCDLBCL-LT as a separate disease entity in the classification system of the European Organization for Research and Treatment of Cancer (EORTC) with differentiation between PCDLBCL-LT and PCFCL-LC being primarily based on clinical criteria (leg vs. non-leg presentation) [4, 5]. With the introduction of the World Health Organization (WHO)—EORTC classification system in 2005, differentiation between both conditions was no longer primarily based on clinical criteria, but on a combination of morphological, immunophenotypical, and clinical criteria [6]. PCFCL-LC shows diffuse infiltrates of large centrocytes (large cleaved cells), no or minimal expression of BCL2 and MUM1, and preferentially presents on the head or trunk. In contrast, PCDLBCL-LT is characterized by the presence of confluent sheets of centroblasts and/or immunoblasts (large non-cleaved cells), strong expression of BCL2 and MUM1, and in more than 80% of cases presents with skin tumors on the leg(s). More recently, expression of IgM, *MYD88* mutations, and loss of *CDKN2A* have been described as new, highly sensitive and/or specific biomarkers favoring a diagnosis of PCDLBCL-LT [7–11]. Using this multiparameter approach, differentiation between PCDLBCL-LT and PCFCL-LC is generally not difficult.

Compared with CLBCL, the classification of nodal/systemic diffuse large B-cell lymphoma (DLBCL) is more complex. Apart from few more specific anatomical-related subgroups, most cases are classified as DLBCL, not otherwise specified (NOS). In contrast to CLBCL, simple clinical and histological criteria to recognize clinically relevant subgroups in DLBCL-NOS are not available. Gene-expression profiling (GEP) of DLBCL-NOS revealed two major molecular subgroups based on their cell-of-origin (COO) with prognostic significance: the germinal center B-cell-like (GCB) subtype and the activated B-cell-like (ABC) subtype, of which GCB-DLBCL shows a superior survival compared with ABC-DLBCL [12]. However, for this “gold standard” method of GEP, fresh-frozen tissue is needed and this is usually not available in routine diagnostics. Therefore, several alternative methods have been developed as surrogates for COO classification in DLBCL-NOS on formalin-fixed and paraffin-embedded (FFPE) tissue. The most commonly used alternative is the immunohistochemistry-based Hans algorithm that evaluates protein expression of CD10, BCL6, and MUM1 for classification of DLBCL as either GCB or non-GCB subtype [13]. Later, a more accurate and precise alternative for conventional microarray-based GEP was introduced with the Lymph2Cx algorithm using the NanoString (nCounter) technology that is suitable for FFPE tissue [14, 15].

In CLBCL, Hoefnagel et al. [16] demonstrated that there are similarities between PCDLBCL-LT and ABC-DLBCL as well as between PCFCL and GCB-DLBCL using conventional array-based GEP. The aim of the present study was to investigate the discriminative performance of COO classification using the Hans and Lymph2Cx algorithms in a defined group of PCFCL-LC and PCDLBCL-LT.

## Materials and methods

### Case selection

Skin biopsies of 66 patients with a CLBCL, including 51 patients with PCDLBCL-LT and 15 patients with PCFCL-LC, were selected from the archives of the Departments of Pathology of the Leiden University Medical Center (LUMC), The Netherlands (*n* = 60), and the University Hospitals Leuven (UZL), Belgium (*n* = 6). Only cases with diffuse infiltrates that were predominantly composed of large B cells were included. Clinical information and follow-up data were collected from the Dutch Registry of Cutaneous Lymphoma and/or from medical records. Thirty-five patients with PCDLBCL-LT and 11 patients with PCFCL-LT were reported in previous studies analyzing immunohistochemical and genetic markers [8, 17]. FFPE skin biopsies were centrally reviewed by AS, PJ, and RW and all patients were diagnosed according to the criteria of the 2016 revision of the WHO classification and the 2018 update of the WHO-EORTC classification [1, 18]. At time of diagnosis, presence of extracutaneous disease was excluded by standard staging procedures, consisting of a fluorodeoxyglucose positron emission tomography-computed tomography (FDG PET-CT) scan or a CT scan in combination with a bone marrow biopsy. 

### Immunohistochemistry

Immunohistochemistry (IHC) was performed with the Dako Autostainer Link 48, according to the manufacturer’s recommendations, for the antibodies CD10 (clone 56C6 from Dako, diluted 1:20), BCL6 (clone PG-B6p from Invitrogen, diluted 1:100), MUM1 (clone MUM1p from Dako, diluted 1:100), BCL2 (clone 124 Dako, diluted 1:80), and IgM (polyclonal, from Dako, diluted 1:500). The markers were scored positive in case of expression in ≥ 30% of the tumor cells for CD10, BCL6, and MUM1, and in ≥ 50% of the tumor cells for BCL2 and IgM.

### Molecular analysis

For molecular analysis, DNA and mRNA were isolated with the Tissue Preparation System (TPS) robot (Siemens Healthcare Diagnostics) from 10 μm microdissected sections of FFPE tissue blocks that contained ≥ 60% tumor cells, as described previously [19]. For GEP with NanoString, in total, ~ 150 ng (range 100 to 200 ng) of RNA per sample was used. The resulting raw counts obtained by NanoString gene-expression analysis were uploaded at the Lymphoma/Leukaemia Molecular Profiling Project (LLMPP) website for COO categorization (https://llmpp.nih.gov/LYMPHCX/index.shtml) [15]. The Lymph2Cx panel evaluates the expression of 20 genes: 7 ABC-related genes (*TNFRSF13B*, *LIMD1*, *IRF4*, *CREB3L2*, *PIM2*, *CYB5R2*, *RAB7L1*, and *CCDC50*), 8 GCB-related genes (*MME*, *SERPINA9*, *ASB13*, *MAML3*, *ITPKB, MYBL1*, and *S1PR2*), and 5 housekeeping genes for normalization (*R3HDM1*, *WDR55*, *ISY1*, *UBXN4*, *TRIM56*). Cases with a normalization of the housekeeping genes below 20 were excluded from further analysis because of poor quality of the sample (*n* = 0). The weight of all genes was analyzed and a probability score between 0 and 1 was generated, as described by Scott et al. [15]. A probability score ≤ 0.1 indicated classification as GCB, 0.11 to 0.89 as unclassified/intermediate (UI), and ≥ 0.90 as ABC.

In addition to GEP, the general molecular profile was determined via targeted next-generation sequencing (tNGS) with the LYMFv1 panel including 52 B-cell lymphoma-related genes, according to the methods described in a previous study [20]. The complete molecular profile of PCDLBCL-LT and PCFCL-LC was included in Supplemental Fig. 1. For the purpose of the current study, only the mutational status of *MYD88* and *CD79B* and loss of *CDKN2A* were reported.

**Fig. 1:**
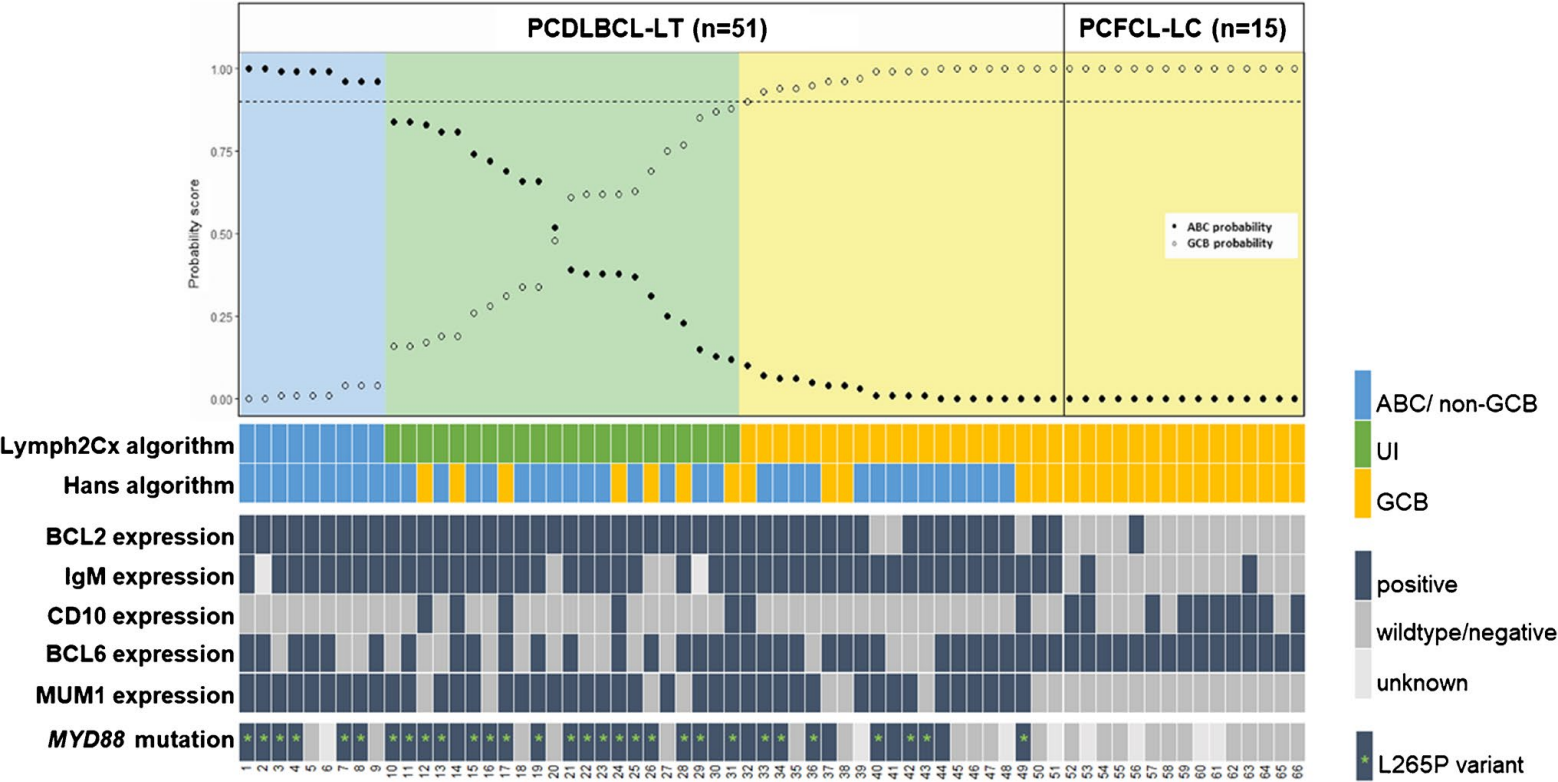
OncoPrint of cell-of-origin (COO) classification, immunohistochemistry, and *MYD88* status in 51 cases with primary cutaneous diffuse large B-cell lymphoma, leg type (PCDLBCL-LT) and 15 cases of primary cutaneous follicle center lymphoma with a diffuse population of large cells (PCFCL-LC). The Hans and Lymph2Cx algorithms were fully concordant in the PCFCL-LC cases (15/15; 100%) but were discordant in the majority (36/51; 71%) of the cases with PCDLBCL-LT. The majority of PCDLBCL-LT cases expressed BCL2 (94%), IgM (94%), and MUM1 (82%), while expression of BCL2 (7%), IgM (7%), and MUM1 (0%) was absent or only rarely present in PCFCL-LC. Mutations in *MYD88* were detected in 36 of 47 (77%) of PCDLBCL-LT but in none of 11 (0%) PCFCL-LC cases. There was no statistically significant difference for the frequency of BCL2 or IgM expression and *MYD88* mutations between the COO subgroups as defined by Hans or Lymph2Cx in PCDLBCL-LT. Remark: A probability score ≤ 0.1 indicated classification as GCB, 0.11 to 0.89 as unclassified, and ≥ 0.90 as ABC.[15]. Abbreviations: PCDLBCL-LT, primary cutaneous diffuse large B-cell lymphoma, leg type; PCFCL-LC, primary cutaneous follicle center lymphoma with a diffuse population of large cells; ABC, activated B-cell-like subtype; GCB, germinal center B-cell-like subtype; UI, unclassified/intermediate. *MYD88 non-L265P mutations consisted of S234N (*n* = 4), Y240S (*n* = 1), and M232T (*n* = 1)

### Statistical analysis

Comparison of the characteristics between the COO subgroups was performed with the *χ*^2^ test for categorical variables and the Kruskal–Wallis test for continuous variables. The overall survival (OS) and DSS were estimated with the cumulative proportion surviving at the end of 5 years of follow-up duration. In time-to-event analysis, the starting point was the date of the histological diagnosis. An event was defined as death by any cause for OS and death by lymphoma/lymphoma-related death for DSS. Patients without an event at the last time of follow-up were censored. Risk groups were compared with the log-rank test. Median follow-up duration was calculated with the reverse Kaplan–Meier method [21]. Statistical analyses were performed with IBM SPSS Statistics 25. A *p*-value of < 0.05 was considered statistically significant.

## Results

The clinical characteristics and follow-up data, immunohistochemistry, genetic alterations, and the results of COO classification of the 51 PCDLBCL-LT and 15 PCFCL-LC are presented in Table 1, Fig. 1, and Supplemental Fig. 1.

**Table 1:** Patient characteristics and overview of results of cell-or-origin classification with the Hans and Lymph2Cx algorithms in patients with primary cutaneous large B-cell lymphomas

	PCFCL-LC	PCDLBCL-LT							
Characteristics	All cases/all GCB (*n* = 15)	All cases (*n* = 51)	Lymph2Cx			*p*-value^a^	Hans		*p*-value^a^
			ABC (*n* = 9)	UI (*n* = 22)	GCB (*n* = 20)		non-GCB (*n* = 38)	GCB (*n* = 13)	
Female gender, *n* (%)	4 (27)	26 (51)	6 (67)	9 (41)	11 (55)	0.39	23 (61)	3 (23)	0.020
Median age at diagnosis, year (range)	58 (37–67)	78 (47–92)	80 (47–86)	77 (49–86)	79 (53–92)	0.77	79 (47–92)	77 (56–86)	0.27
Leg(s) involved at diagnosis, *n* (%)	1 (7)^b^	43 (84)	7 (78)	19 (86)	17 (85)	0.83	32 (84)	11 (85)	0.97
Initial therapy						0.17			0.32
Local radiotherapy	15 (100)	23 (45)	1 (11)	12 (55)	10 (50)		15 (39)	8 (62)	
(Immuno)chemotherapy^c^	0	26 (51)	8 (89)	9 (41)	9 (45)		21 (55)	5 (38)	
None^d^	0	2 (4)	0	1 (5)	1 (5)		2 (5)	0	
Median follow-up duration, years (range)	7.0 (4.3–16.1)	11.3 (0.17–16.3)	11.3 (0.6–11.3)	6.9 (0.6–13.4)	12.1 (0.17–16.3)	0.58	9.9 (0.17–16.3)	11.3 (2.0–13.0)	0.61
5-year overall survival, %	100	48	29	48	58	0.38	43	59	0.43
5-year disease-specific survival, %	100	59	33	61	69	0.36	51	81	0.14
Immunohistochemistry									
BCL2	1 (7)	48 (94)	9 (100)	22 (100)	17 (85)	0.09	36 (95)	12 (92)	0.75
IgM	1 (7)	46 (94)^f^	8 (89)^e^	18 (82)	20 (100)	0.12	34 (89)^f^	12 (92)	0.78
CD10	9 (60)	7 (14)	0	5 (23)	2 (10)	0.21	0	7 (54)	0.000
BCL6	15 (100)	34 (67)	6 (67)	12 (55)	16 (80)	0.22	22 (58)	12 (92)	0.023
MUM1	0	42 (82)	9 (100)	18 (82)	15 (75)	0.26	36 (95)	6 (46)	0.000
Genetic profile									
*MYD88* mutation	0^ h^	36 (77)^h^	6 (75)^e^	19 (86)	11 (55)^g^	0.28	26 (74)^g^	10 (83)^e^	0.52
Hans algorithm						0.15			
Non-GCB	0	38 (75)	9 (100)	15 (68)	14 (70)		38 (100)	NA	NA
GCB	15 (100)	13 (25)	0	7 (32)	6 (30)		NA	13 (100)	
Lymph2Cx algorithm						NA			0.15
ABC	0	9 (18)	9 (100)	NA	NA		9 (24)	0	
UI	0	22 (43)	NA	22 (100)	NA		15 (41)	7 (50)	
GCB	15 (100)	20 (39)	NA	NA	20 (100)		14 (37)	6 (46)	

Abbreviations: *PCFCL-LC*, primary cutaneous follicle center lymphoma with a diffuse population of large cells; *PCDLBCL-LT*, primary cutaneous diffuse large B-cell lymphoma, leg type; *GCB*, germinal center B-cell-like; *ABC*, activated B-cell-like; *UI*, unclassified/intermediate; *NA*, not applicable

^a^*χ*^2^ test for categorical data, the Mann–Whitney *U* or Kruskal Wallis test for continuous variables in case of the Hans algorithm (2 groups) or the Lymph2Cx algorithm (3 groups), respectively, and log-rank for survival analysis. Bold values are statistically significant

^b^This patient presented with skin lesions on the right arm and left leg

^c^(Immuno)chemotherapy consisted of R-CHOP, CHOP, or (R)CHOP-like regimens, such as R-CEOP. From the patients who received (immuno)chemotherapy, 21 (81%) patients received regimens with rituximab and five (19%) patients, all diagnosed between 2001 and 2005, received regimens without rituximab

^d^No therapy was given because of spontaneous remission of the skin lesions (*n* = 1) and sudden unrelated death (*n* = 1)

^e^Data is missing in one case

^f^Data is missing in two cases

^g^Data is missing in three cases

^h^Data is missing in four cases

### Clinical characteristics

Patients with PCDLBCL-LT included 26 females and 25 males. The median age at diagnosis was 78 (range, 47 to 92) years. Forty-three of 51 (84%) patients presented with skin lesions on one or both legs. In the remaining patients, disease was located on the upper lip (*n* = 1), cheek (*n* = 1), abdomen (*n* = 1), arm (*n* = 3), hip (*n* = 1), and penis (*n* = 1). Initial therapy with multiagent chemotherapy (*n* = 21 with rituximab and *n* = 5 without rituximab) or radiotherapy (*n* = 23) resulted in a complete remission in 48 of 49 (98%) cases. In two patients, no treatment was given because of spontaneous remission of the skin lesions (*n* = 1) or sudden unrelated death (*n* = 1). After a median follow-up of 11.3 (range, 0.17 to 16.3) years, 20 patients were still alive with (*n* = 8) or without (*n* = 12) evidence of disease, 16 patients had died of lymphoma, and 15 patients had died of unrelated disease. At 5 years, the overall survival was 48% and the disease-specific survival was 59%.

Patients with PCFCL-LC included four females and 11 males with a median age at diagnosis of 58 (range, 37 to 67) years. Fourteen of 15 (93%) patients presented with (localized) skin lesions in the head-and-neck region and/or trunk. The remaining patient presented with skin lesions on the right arm and left leg. Initial treatment consisted of radiotherapy and resulted in a complete remission in all patients. After a median follow-up of 7.0 (range, 4.3 to 16.1) years, all 15 patients were alive with (*n* = 8) or without (*n* = 7) evidence of disease.

### Histopathology and immunohistochemistry

PCDLBCL-LT showed diffuse infiltrates of centroblasts and/or immunoblasts (large non-cleaved cells), which expressed BCL2 in 48 of 51 (94%), MUM1 in 42 of 51 (82%), and IgM in 46 of 49 (94%) cases (Fig. 1). PCFCL-LC consisted of diffuse populations of large centrocytes (large cleaved cells). In contrast to PCDLBCL-LT, BCL2 and IgM were expressed in only one of 15 (7%) cases each, while MUM1 staining was negative in all cases.

#### Hans algorithm

In PCDLBCL-LT, the immunohistochemical markers of the Hans algorithm, CD10, BCL6, and MUM1, showed expression in 7 of 51 (14%), 34 of 51 (67%), and 42 of 51 (82%) patients, respectively (Fig. 1). As most cases expressed MUM1 and were negative for CD10, the majority (38/51; 75%) was classified as non-GCB. In the remaining 13 cases (25%), GCB classification was attributed to the expression of CD10 in seven patients and isolated expression of BCL6 in six patients. Comparison between PCDLBCL-LT cases classified as either non-GCB or GCB showed no differences in the expression of BCL2 and IgM as well as in survival.

In the PCFCL-LC group, all patients (15/15; 100%) were classified as GCB subtype. This was attributed to both CD10 and BCL6 expression in eight patients and to isolated BCL6 expression in seven patients. None of the cases expressed MUM1.

### Gene-expression profiling

#### Lymph2Cx algorithm

In contrast to the Hans algorithm, COO classification of the PCDLBCL-LT patients with the Lymph2Cx panel identified only nine of 51 (18%) patients as ABC subtypes (Fig. 1). The other cases were classified as GCB (20 of 51; 39%) or as UI (22 of 51; 43%). The Lymph2Cx prediction scores ranged between 0.00 and 1.00. Regarding the patient characteristics, immunohistochemistry, *MYD88* and *CD79B* mutations, loss of *CDKN2A*, and survival of the PCDLBCL-LT patients, there were no statistically significant differences between the three COO subgroups. All nine patients in the ABC subgroup were also classified as non-GCB by the Hans algorithm (100% concordance), but in the GCB category, classification of only six of 20 (30%) patients was concordant. The UI category consisted of a mixture of cases classified as non-GCB (15/22; 68%) and GCB (7/22; 32%) by Hans. 

In the PCFCL-LC group, COO classification with the Lymph2Cx algorithm was fully concordant with the Hans algorithm and all 15 patients were classified as GCB subtypes. Their prediction scores were all determined at 0.00.

### Genetic profile

The results of mutational analysis with our 52-gene panel are presented in Supplementary Fig. 1 for the PCDLBCL-LT and Supplementary Fig. 2 for the PCFCL-LC. Thirty-six of 47 (77%) PCDLBCL-LT patients harbored a *MYD88* mutation, including the hotspot L265P in 30 (83%) of these patients, and 22 of 47 (47%) patients had a *CD79B* mutation, with the hotspot Y196 mutation in 13 (59%) of these cases. Loss of *CDKN2A* was identified in 29 of 47 (62%) cases. In our cohort of PCDLBCL-LT, *MYD88*, and/or *CD79B* mutations as well as loss of *CDKN2A* were present in all COO subtypes, either defined by Hans or the Lymph2Cx algorithm, without a statistical difference (data not shown). None of the successfully analyzed PCFCL-LC patients (*n* = 11) harbored a *MYD88* or *CD79B* mutation.

## Discussion

Diagnosis of CLBCL is primarily made on a combination of the clinical presentation and the morphology and immunophenotype of the tumor cells (supported by specific molecular markers). In order to determine the discriminative performance of COO classification in CLBCL, this study describes the results of the IHC-based Hans algorithm and the GEP-based Lymph2Cx algorithm in 66 patients with a CLBCL, including 51 patients with PCDLBCL-LT and 15 patients with PCFCL-LC.

All cases of PCFCL-LC were uniformly classified as GCB by both the Hans algorithm and the Lymph2Cx algorithm. These results correspond to literature with GCB classification of PCFCL-LC in 23 of 25 (92%) cases using Hans and in all ten (100%) PCFCL cases with spindle-cell morphology using NanoString technology [22, 23].

In contrast to the homogeneous results in PCFCL-LC, the results of COO classification in PCDLBCL-LT patients were heterogeneous. Using Hans, 75% of the PCDLBCL-LT patients classified as non-GCB and 25% as GCB. However, using the Lymph2Cx algorithm, only 18% of the cases was classified as ABC, while 39% was determined as GCB and 43% as UI.

Regarding the Hans algorithm, our results correspond roughly to a study reporting non-GCB classification in 14 of 23 (61%) PCDLBCL-LT patients [24]. In another study, however, all 32 PCDLBCL-LT cases (100%) had a non-GCB phenotype [22]. In this latter study, CD10 expression, a key marker of the Hans algorithm, was used as an exclusion criterion for diagnosis of PCDLBCL-LT. Although CD10 expression is uncommon in PCDLBCL-LT, it has never been used as an exclusion criterion for diagnosis of PCLDBCL-LT [18, 25]. In our cohort of PCDLBCL-LT, all CD10-positive cases showed the characteristic features of PCDLBCL-LT, including presentation on the leg(s), round-cell morphology, lack of (residual) follicular dendritic cell-networks, expression of BCL2 and MUM1 in all but one case, IgM expression in all cases, and *MYD88* mutations in all cases. In addition, there was no difference in survival between the CD10-positive and CD10-negative PCDLBCL-LT cases (OS: 57% vs. 46%, log-rank 0.69; DSS: 69% vs. 57%, log-rank *p*-value 0.67). Therefore, there is no reason to use CD10 expression for exclusion as PCDLBCL-LT.

Regarding the Lymph2Cx algorithm, no studies specifically using this technique in PCDLBCL-LT patients have been published so far. Interestingly, the percentage of ABC-classified cases (18%) of PCDLBCL-LT in our cohort is much lower than expected based on the similarities in GEP of PCDLBCL-LT with ABC-DLBCL and PCFCL-LC with GCB-DLBCL, as described by Hoefnagel et al. [16]. Also, the immune phenotype of PCDLBCL-LT is characterized by protein expression of the activated B-cell markers BCL2, MUM1, and IgM in the vast majority of the patients. Accordingly, the genetic profile is dominated by NFκB-activating mutations, predominantly in *MYD88* and *CD79B*, which are enriched in ABC-DLBCL and, especially, primary central nervous system lymphoma (PCNSL), primary testicular lymphoma (PTL), and intravascular large B-cell lymphoma [26–28]. In contrast, the GCB marker BCL6 is commonly expressed [2] and a significant subgroup of PCDLBCL-LT harbors *MYC* rearrangements [17, 29], generally seen as an early GC event [30]. In PCNSL and PTL, 60 to 96% is classified as non-GCB by the Hans algorithm, corresponding to our results (75%) in PCDLBCL-LT [31–34]. However, Bödör et al. [31] identified a much higher percentage of ABC cases defined by Lymph2Cx in 77 PCNSL cases (81%) compared with PCDLBCL-LT in our study (18%). On the other hand, Montesinos et al. [35] also demonstrated heterogeneity in GEP profiles in 21 PCNSL cases, as 24% classified as ABC, 33% as GCB, and 43% as UI using the COO algorithm developed by Wright et al. [36]. In addition, a study using microarray-based GEP in 23 PCNSL cases demonstrated overlapping expression of germinal-center and activated B-cell genes [37]. In line with these findings, recent studies in DLBCL-NOS also demonstrated that binary classification into ABC or GCB does not correspond to the more complex biology as identified by transcriptomics and that recurrent genetic alterations are identified beyond the established COO subtypes [38–40].

The discrepancy in results of COO classification in PCDLBCL-LT may partly be clarified by the significantly different techniques and diverse selection of genes used for classification. The analysis of Hoefnagel et al. [16] was based on 43 discriminating genes, of which only 3 genes (*IRF4*, *ITPKB*, and *PIM2*) overlapped with the genes in the Lymph2Cx panel. In addition, their study did not address the UI category, as was determined in 43% of our PCDLBCL-LT cases. Nevertheless, the poor correspondence and high percentage (43%) of UI may suggest a different biology of PCDLBCL-LT compared with DLBCL-NOS for which these algorithms were originally developed. Future studies need to investigate this observation with more comprehensive transcriptomic analysis, such as the EcoTyper, as recently described by Steen et al. [40].

In our cohort of PCDLBCL-LT, *MYD88* and/or *CD79B* mutations as well as survival did not statistically differ between the COO subtypes defined by both algorithms. This corresponds to PCNSL, as Bödör et al. [31] also reported equal distribution of *MYD88* and/or *CD79B* mutations and no differences in survival between the COO subgroups.

While our results showed that classification as non-GCB with Hans or ABC/UI with Lymph2Cx strongly favors a diagnosis of PCDLCBL-LT, a GCB outcome does not differentiate between PCFCL-LC and PCDLBCL-LT. Use of COO algorithms for this purpose should, therefore, be interpreted with caution, as incorrect interpretation may have far stretching clinical implications. For instance, PCDLBCL-LT as defined by the 2016 revised WHO classification or the 2018 update of the WHO-EORTC classification but categorized as GCB by either the Hans or Lymph2Cx algorithm may be misdiagnosed as PCFCL-LC and, subsequently, treated incorrectly as a low-grade CLBCL with radiotherapy instead of immuno-polychemotherapy.

In conclusion, our data confirm the GCB-subtype of PCFCL-LC but show that PCDLBCL-LT cases classify along the whole COO spectrum of DLBCL-NOS using the Hans and Lymph2Cx algorithms. In contrast to systemic DLBCL-NOS, the clinical relevance of COO classification in CLBCL using these algorithms is limited and cannot be used as an alternative for the current multiparameter approach in differentiation of PCDLBCL-LT and PCFCL-LC.

## Supplementary Information

Below is the link to the electronic supplementary material.

Supplementary file1 (PDF 429 KB)
