# Supplementary material for: Cell-of-origin classification using the Hans and Lymph2Cx algorithms in primary cutaneous large B-cell lymphomas
Source: Virchows Arch. 2022 Jan 14;480(3):667–75. doi: 10.1007/s00428-021-03265-5 (PMC8990933; doi:10.1007/s00428-021-03265-5)
Supplement: Supplementary file 1 — Supplementary file1 (PDF 429 KB) [file 428_2021_3265_MOESM1_ESM.pdf]

SUPPLEMENTAL INFORMATION

Cell-of-origin classification using the Hans and Lymph2Cx algorithms in primary cutaneous large B-cell lymphomas

Virchows Archives

Anne M. R. Schrader[1], Ruben A. L. de Groen[2], Rein Willemze[3], Patty M. Jansen[1], Koen D. Quint[3], Tom van Wezel[1], Ronald van Eijk[1], Dina Ruano[1], Cornelis P. Tensen[3], Esther Hauben[4], F.J.S.H. (Sherida) Woei-A-Jin[5], Anne M. Busschots[6], Anke van den Berg[7], Arjan Diepstra[7], Maarten H. Vermeer[3], and Joost S. P. Vermaat[2]

[1]Department of Pathology, [2]Department of Hematology, and [3]Department of Dermatology, Leiden University Medical Center, Leiden, The Netherlands; and [4]Department of Pathology, [5]Department of General Medical Oncology, [6]Department of Dermatology, University Hospitals Leuven, Leuven, Belgium, and [7]Department of Pathology, University Medical Center Groningen

Corresponding author:

Anne M. R. Schrader, MD PhD

E-mail address: a.m.r.schrader@lumc.nl

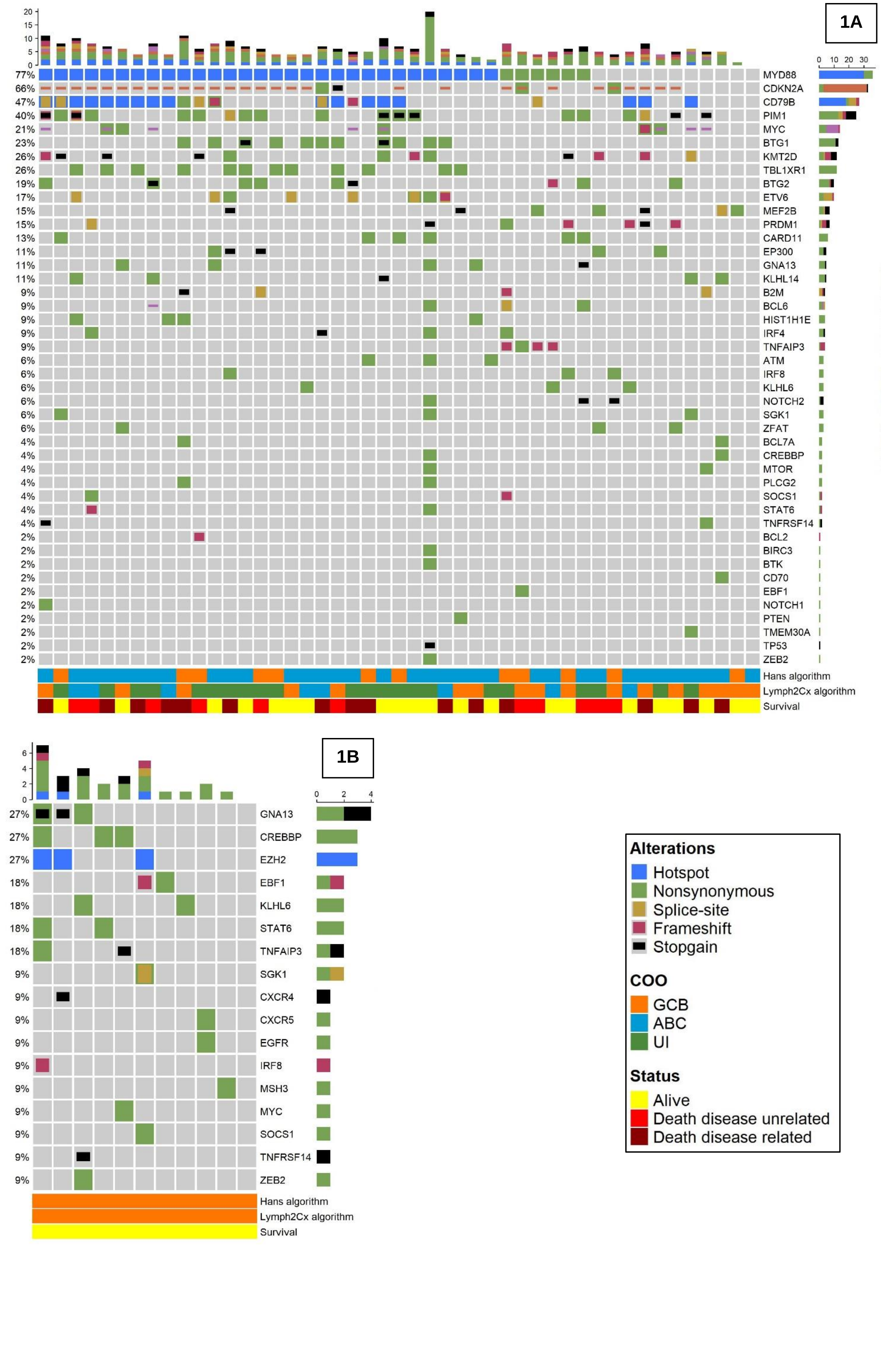
1A
20
15
10
5
0
0 10 20 30
77% MYD88
66% CDKN2A
47% CD79B
40% PIM1
21% MYC
23% BTG1
26% KMT2D
26% TBL1XR1
19% BTG2
17% ETV6
15% MEF2B
15% PRDM1
13% CARD11
11% EP300
11% GNA13
11% KLHL14
9% B2M
9% BCL6
9% HIST1H1E
9% IRF4
9% TNFAIP3
6% ATM
6% IRF8
6% KLHL6
6% NOTCH2
6% SGK1
6% ZFAT
4% BCL7A
4% CREBBP
4% MTOR
4% PLCG2
4% SOCS1
4% STAT6
4% TNFRSF14
2% BCL2
2% BIRC3
2% BTK
2% CD70
2% EBF1
2% NOTCH1
2% PTEN
2% TMEM30A
2% TP53
2% ZEB2
Hans algorithm
Lymph2Cx algorithm
Survival
1B
6
4
2
0
0 2 4
27% GNA13
27% CREBBP
27% EZH2
18% EBF1
18% KLHL6
18% STAT6
18% TNFAIP3
9% SGK1
9% CXCR4
9% CXCR5
9% EGFR
9% IRF8
9% MSH3
9% MYC
9% SOCS1
9% TNFRSF14
9% ZEB2
Hans algorithm
Lymph2Cx algorithm
Survival
Alterations
Hotspot
Nonsynonymous
Splice-site
Frameshift
Stopgain
COO
GCB
ABC
UI
Status
Alive
Death disease unrelated
Death disease related

Supplemental Figure 1. OncoPrints of the molecular alterations identified with the LYMFv1 targeted next-generation sequencing panel (52 B-cell lymphoma-relevant genes) and fluorescence *in situ* hybridization for *MYC* for 47 patients with PCDLBCL-LT (1A) and 11 patients with PCFCL-LC (1B). The majority of PCDLBCL-LT harbored a mutation in NFκB-related genes, i.e. *MYD88* (77%), *CD79B* (47%), and *PIM1* (40%). Other recurrent alterations were loss of *CDKN2A* (62%) or a rearrangement of *MYC* (17%). Cell-of-origin (COO) classification assigned the majority (75%) of PCDLBCL-LT to the non-GCB subtype with the Hans algorithm and mostly to UI (43%) or GCB (39%) with the Lymph2Cx algorithm. The cohort of PCFCL-LC demonstrated frequent mutations in *GNA13* (27%), *CREBBP* (27%), and *EZH2* (27%). All PCFCL-LC cases were classified as GCB by both Hans and Lymph2Cx. All PCFCL-LC patients were alive at last follow-up compared with only 45% of the PCDLBCL-LT patients; 32% of the PCDLBCL-LT patients died disease-related and 23% died disease-unrelated.

Abbreviations: COO, cell-of-origin; GCB, germinal center B-cell-like subtype; ABC, activated B-cell-like subtype; UI, unclassified/intermediate.
